# Microvascular changes on optical coherence tomography angiography after rhegmatogenous retinal detachment vitrectomy with silicone tamponade

**DOI:** 10.1371/journal.pone.0248433

**Published:** 2021-03-12

**Authors:** Ji Hye Lee, Young Gun Park

**Affiliations:** Department of Ophthalmology and Visual Science, Seoul St. Mary’s Hospital, College of Medicine, The Catholic University of Korea, Seoul, Korea; Massachusetts Eye & Ear Infirmary, Harvard Medical School, UNITED STATES

## Abstract

**Purpose:**

We aimed to evaluate microvascular changes on optical coherence tomography angiography (OCTA) in patients with rhegmatogenous retinal detachment (RRD) who underwent silicone oil (SO) tamponade and compare changes according to macular involvement.

**Methods:**

This retrospective study included 48 patients with unilateral RRD who underwent vitrectomy and SO tamponade and were stable after SO removal. Control data were obtained from the fellow healthy eye. Ophthalmic examinations, including best corrective visual acuity (BCVA) and OCTA, were conducted. Differences in vascular density (VD) in different sections of the macula and differences in the foveal avascular zone (FAZ) were analyzed between the affected eyes and control eyes. Subgroup analyses according to macular involvement were performed.

**Results:**

Baseline BCVA and duration of SO tamponade were associated with postoperative BCVA (p<0.001, p = 0.03, respectively). The average VD in the deep capillary plexus (DCP) and the VD of the nasal parafoveal area in both the superficial capillary plexus (SCP) and the DCP decreased relative to those in the control eyes (p = 0.026, p = 0.028, and p = 0.031, respectively). The FAZ area in the DCP and in the SCP also increased when compared with that in the controls (p = 0.043, p = 0.002, respectively). In addition, the macular-off RRD group had lower VD in the nasal parafoveal area of the DCP than the macular-on RRD group.

**Conclusion:**

SO tamponade could cause microvascular changes, especially in the nasal parafoveal area. The macular-off RRD group were affected more than the macular-on RRD group.

## Introduction

Rhegmatogenous retinal detachment (RRD) is an important cause of blindness worldwide and occurs in 10,000 people per year [1]. Silicone oil (SO) has been widely used as a long-term intraocular tamponade for treating complex retinal detachments. The performance of an intraocular tamponade reduces recurrence and prevents proliferative retinopathy [2, 3].

Although SO is an effective intraocular tamponade due to its high viscosity and high surface tension, it has been associated with cataract progression, increased intraocular pressure, emulsified oil droplets, and band keratopathy [4]. Unexplained vision loss has been occasionally reported after SO injection [5, 6], and Scheerlinck et al. [7] showed that the incidence of SO-related visual loss was 30% for the duration of the tamponade.

Recently, several studies have reported changes related to SO tamponade using optical coherence tomography (OCT) and microperimetry. These changes are widely assumed to be linked to SO-induced toxicity and ischemia, which result in damage to ganglion cells and the inter-nuclear synapsis [8, 9].

OCT angiography (OCTA) is a noninvasive imaging modality used to display retinal and choroidal blood vessels. It can assess the vascular density, structures of the retinal bloodstream, and figures of the foveal avascular zone (FAZ). Microvascular changes related to retinal detachment have been described in current OCTA researches. Some investigators have reported microvascular changes in the superficial capillary plexus (SCP) and in the deep capillary plexus (DCP) in patients with RRD after vitrectomy using SO or gas tamponade [10–12]. In contrast, others have suggested that the macular microvasculature remains intact [13, 14]. However, there is a lack of research on quantitative microvascular changes in the different retinal vascular layers in patients with RRD who underwent SO tamponade depending on macular involvement.

Therefore, this study aimed to evaluate retinal microvascular changes in the FAZ and parafoveal segments after SO tamponade and compare these changes, according to macular involvement, between macular-on and macular-off RRD using OCTA.

## Materials and methods

### Participants

This study was a retrospective review of consecutive cases. Patients who presented to the Department of Ophthalmology of Seoul St. Mary’s Hospital in Seoul, Korea, between January 2018 and January 2020 with a confirmed diagnosis of RRD and underwent pars plana vitrectomy using SO tamponade were included. The healthy control data were extracted from the patients’ unaffected fellow eye. The study followed by tenets of the Declaration of Helsinki and was approved by the Institutional Review Board of Seoul St. Mary’s Hospital and the Catholic University of Korea. The need to obtain informed patient consent was waived due to the retrospective study design.

The inclusion criteria were the successful repair of primary RRD following surgery with SO tamponade and a minimum of 3 months of follow-up after SO removal. The exclusion criteria were traumatic or tractional RD, the co-occurrence of any other ocular disease related to ocular circulation (e.g., macular degeneration, glaucoma, and axial length > 26 mm), history of previous intraocular surgery, and severe media opacity.

All patients underwent baseline ophthalmic examinations, which included best-corrected visual acuity (BCVA), slit-lamp examination, color fundus photography, swept-source OCT, and OCTA imaging (DRI OCT Triton; Topcon, Tokyo, Japan) on the day or the day before the operation. We assessed the postoperative scans, which were obtained 3 months after silicone oil removal, and the findings were compared. BCVA was converted into the logarithm of the minimum angle of resolution (logMAR) for statistical analysis.

### Optical coherence tomography angiography

OCTA scans were obtained with DRI OCT Triton (Topcon) for a 4.5 × 4.5-mm square centred upon the fovea. Patients having low-quality images with a signal strength index of under 50 were excluded. OCTA images were evaluated using automatic segmentation. We analyzed the vascular density (VD) of the superficial and deep retinal vascular zones (i.e., the SCP and the DCP) within the 1- and 3-mm inner and outer circles, respectively, using the built-in software program. The FAZ area was measured by manual delineation and was calculated.

### Surgical procedure

A 3-port 25-gauge vitrectomy (Constellation^®^ Vision system, Alcon Laboratories Inc., Fort Worth, TX, USA) was performed by a single surgeon (YGP). Briefly, after vitreous removal, subretinal fluid drainage and endolaser were performed, and SO (Arciolane 5500; Arcadophta, Toulouse, France) was injected into the vitreous cavity. After at least 3 months of SO tamponade, the SO was removed with confirmation of retinal reattachment [15].

### Statistical analysis

A Mann–Whitney U test was used to compare the parameters between the cases and the controls. Spearman’s rank correlation analysis was used for a univariate analysis, followed by a stepwise multivariate analysis. Data are provided as means ± standard deviations for quantitative data and as numbers (percentages) for qualitative data. All analyses were performed using a commercial software (SPSS version 22.0; IBM, Armonk, NY, USA). P < 0.05 was considered significant.

## Results

### Baseline characteristics

Forty-eight patients (27 men and 21 women) were finally included in the study. Data of their affected and healthy fellow eyes were used as case and control data, respectively. The mean age of patients was 57.6 ± 13.1 years. Forty of 48 eyes (83.3%) exhibited mild cataract (2.02 ± 0.78) based on the lens opacity classification [LOCSIII] scale [16] and underwent combined cataract operations on the affected eye. Twenty-five patients (52.1%) demonstrated macular involvement in the condition (macular-off RRD), and the duration of RRD before surgery was 8.82 ± 5.42 days. The mean duration of SO tamponade was 4.73 ± 2.1 months. Baseline information can be found in Table 1.

**Table 1:** Baseline characteristics.

Patient characteristics	Cases
Patients	48
Mean age (years)	57.6 ± 13.1
Male/female	27/21
Duration of RRD before surgery (days)	8.82 ± 5.42
Macular involvement	25 eyes (52.1%)
Combined cataract surgery	40 eyes (83.3%)
Preoperative BCVA (log MAR)	1.46 ± 0.07
Duration of SO tamponade (months)	4.73 ± 2.1

RRD, rhegmatogenous retinal detachment; BCVA, best-corrective visual acuity; logMAR, logarithm of the Minimum Angle of Resolution; SO, silicone oil.

### Change in BCVA

The mean BCVA changed from 1.46 ± 0.07 logMAR at baseline to 0.65 ± 0.42 logMAR 3 months after SO removal (p <0.05). In the multivariate analysis, baseline BCVA and duration of SO tamponade were significantly associated with postoperative BCVA among all preoperative factors (ß = -0.745, p <0.001; ß = -0.180, p = 0.03) (Table 2).

**Table 2:** Univariate and multivariate linear regression analysis for postoperative visual acuity.

Variable	Univariate analysis		Multivariate Analysis	
	ß ± SE	P value	ß ± SE	P value
Age	-0.184 ± 0.943	0.178		
Male / Female	0.038 ± 0.152	0.814		
Duration of RRD before surgery (days)	0.09 ± 0.762	0.543		
Macular involvement	-0.041 ± 0.015	0.762		
Combined cataract surgery	0.11 ± 0.138	0.938		
Preoperative BCVA (log MAR)	-0.608 ± 0.531	0.002	-0.745 ± 0.514	< 0.001*
Duration of SO tamponade (Months)	-0.15 ± 0.703	0.048	-0.18 ± 0.67	0.03*

RRD, rhegmatogenous retinal detachment; BCVA, best-corrective visual acuity; logMAR, logarithm of the Minimum Angle of Resolution; SO, silicone oil;

* statistically significant (p <0.05).

### Changes in the VD and FAZ area

**Fig 1:**
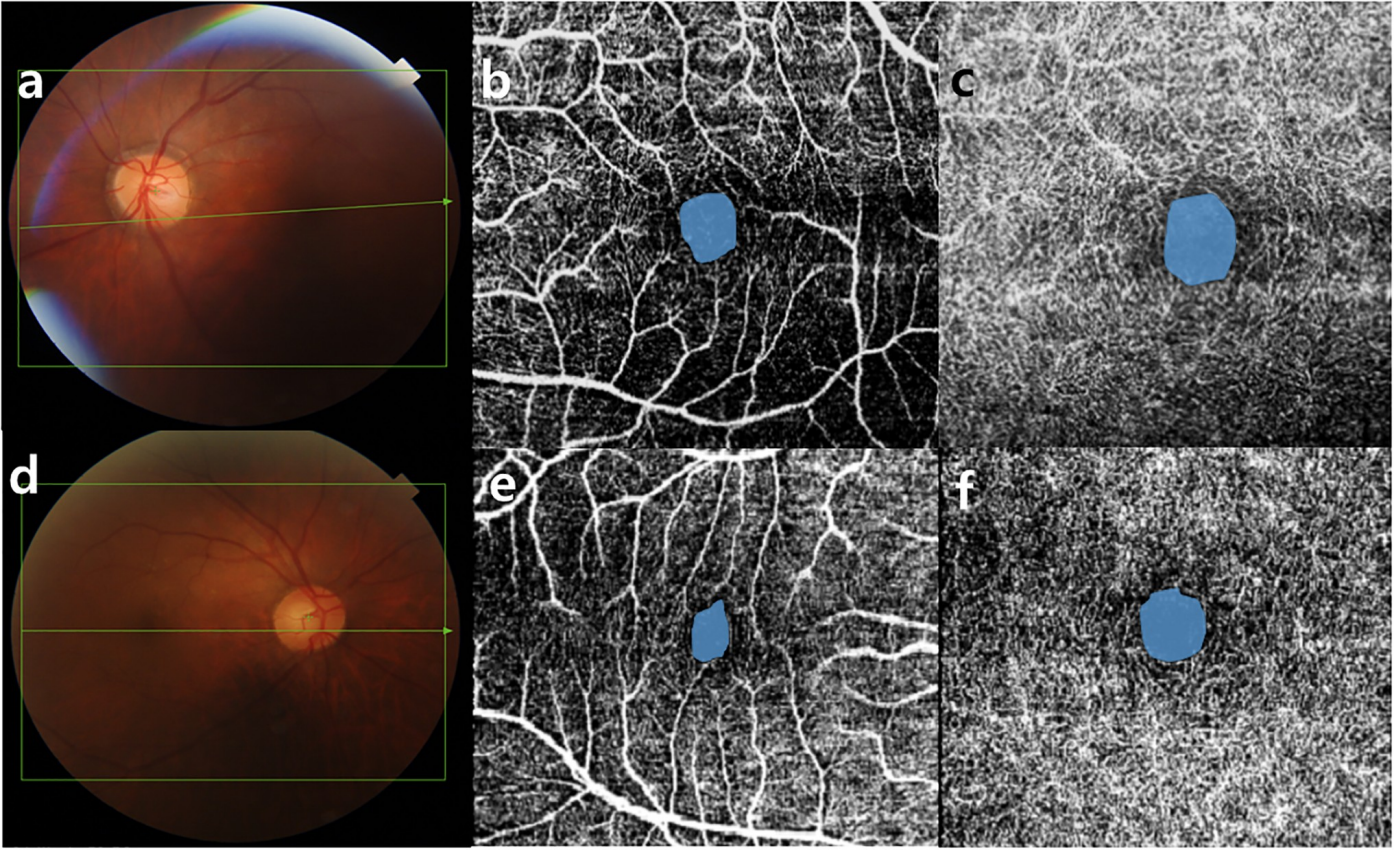
Representative cases of rhegmatogenous retinal detachment repaired with silicone oil tamponade and their fellow healthy eyes. (a-c) A fundus photograph 3 months after retinal detachment repair with silicone oil tamponade and removal is presented. The foveal avascular zone areas of the superficial and deep capillary plexus were enlarged on optical coherence tomography angiography. (d-f) Fundus photography and optical coherence tomography angiography findings of the fellow healthy eye are presented.

The average parafoveal VD of the DCP in the case data was significantly lower than that in the control data (p = 0.026; Fig 1). This was not the case for the SCP, where there was no difference between the cases and controls (p = 0.504). The mean VD of the nasal area in the SCP was significantly different between the two groups (p = 0.028), unlike what was found in other areas (superior/temporal/inferior, p = 0.095, p = 0.059, and p = 0.904, respectively). The mean VD parameters in temporal and nasal segments for the DCP were significantly lower in the cases than in the controls (superior/temporal/inferior/nasal, p = 0.26, p = 0.033, p = 0.733, and p = 0.031, respectively; Table 3). The FAZ areas in the SCP and the DCP were significantly larger in the cases than in the controls (0.28±0.14 mm^2^ vs 0.26±0.13 mm^2^, p = 0.043; 0.56 ± 0.15 mm^2^ vs 0.27 ± 0.12 mm^2^, p = 0.002, respectively; Table 3).

**Table 3:** Retinal microvascular structures evaluated using optical coherence tomography angiography 3 months after silicone oil removal for primary retinal detachment.

	Cases	Controls	p-value
**VD of SCP (%)**			
Average	44.71 ± 3.72	44.50 ± 2.88	0.504
Superior	48.82 ± 7.82	48.82 ± 4.57	0.095
Temporal	40.67 ± 5.66	45.68 ± 3.29	0.059
Inferior	47.38 ± 5.59	49.01 ± 9.01	0.904
Nasal	40.34 ± 5.49	43.27 ± 3.83	0.028*
FAZ area (mm^2^)	0.28 ± 0.14	0.26 ± 0.13	0.043*
**VD of DCP (%)**			
Average	44.65 ± 3.69	45.85 ± 4.43	0.026*
Superior	49.03 ± 8.12	51.92 ± 4.67	0.26
Temporal	41.35 ± 6.72	47.75 ± 3.97	0.033*
Inferior	47.25 ± 9.92	50.31 ± 6.39	0.733
Nasal	40.95 ± 4.41	46.52 ± 4.01	0.031*
FAZ area (mm^2^)	0.30 ± 0.11	0.27 ± 0.12	0.002*

VD, vascular density; SCP, superficial capillary plexus; DCP, deep capillary plexus; FAZ, foveal avascular zone;

* statistically significant (p <0.05).

The association between postoperative BCVA and microvascular OCTA parameters was analyzed. The average parafoveal VD and the nasal VD of the DCP were significantly associated with postoperative BCVA in the multivariate analysis (ß = -0.252, p = 0.003; ß = -0.225, p = 0.01, respectively). A decrease in the average VD of the DCP was identified in patients with SO tamponade. Particularly, the mean VD of the nasal area was significantly lower in the SCP and DCP layers.

### Subgroups analysis by macular involvement

The macular-off RRD group had a lower VD in the nasal parafoveal area of the DCP compared to that in the macular-on RRD group (p = 0.048). The FAZ area of the DCP was also larger in the macular-off than that in the macular-on RRD group (p = 0.009; Table 4).

**Table 4:** Subgroup analysis according to macular involvement.

	Macular-off RRD	Macular-on RRD	p-value
**VD of SCP (%)**			
Superior	46.49 ± 13.97	48.16 ± 6.01	0.09
Temporal	37.98 ± 9.63	40.91 ± 6.43	0.57
Inferior	43.97 ± 12.51	51.32 ± 8.80	0.57
Nasal	37.72 ± 5.52	37.81 ± 9.98	0.29
FAZ area (mm^2^)	0.29 ± 0.04	0.27 ± 0.13	0.89
**VD of DCP (%)**			
Superior	48.12 ± 5.62	51.92 ± 4.67	0.68
Temporal	41.60 ± 7.71	47.75 ± 3.97	0.98
Inferior	48.94 ± 11.8	50.31 ± 6.39	0.31
Nasal	39.23 ± 10.06	39.55 ± 4.36	0.048*
FAZ area (mm^2^)	0.31 ± 0.09	0.29 ± 0.12	0.009*

RRD, retinal detachment; VD, vascular density; SCP, superficial capillary plexus; DCP, deep capillary plexus; FAZ, foveal avascular zone,

*statistically significant (p <0.05).

## Discussion

SOs are valuable tools in cases of retinal detachment vitreoretinal surgery. They are essentially used as intraocular tamponade because of their ability to maintain the attachment. However, long-term tamponade with SO is controversial [17, 18]. We evaluated the changes in retinal microvascular parameters and various factors associated with postoperative visual outcomes in RRD treated with SO tamponade.

Recently, several studies have reported the anatomical and functional changes in eyes that underwent SO tamponade [9, 19]. Roohipoor et al. [20] demonstrated decreased retinal VD as well as decreased retinal thickness in the fovea in macular-off RRD with SO tamponade when compared with that in healthy control eyes. However, they only included patients with macular-off RRD. This entails a limitation regarding parameters related to macular involvement. In addition, they compared the foveal, parafoveal, and choroidal areas. Our study evaluated the changes following RRD repaired with SO and analyzed those changes according to macular involvement. We divided the VD according to the Early Treatment Diabetic Retinopathy Study grid. This procedure is useful for revealing the possible mechanisms of SO-related vision loss.

Although many factors were identified as the predictors of postoperative BCVA in the study cases, baseline BCVA and duration of SO tamponade were the most important predictors of visual outcome. Our study compared OCTA parameters and the area of FAZ between the affected eye of RRD patients with SO tamponade and their control fellow eye, and also assessed the correlation of said parameters with visual outcomes. The average VD of the DCP, but not that of the SCP, was significantly lower in cases than in controls (p = 0.026). This may be secondary to ischemic damage to the retina. Studies on other ischemic retinal diseases have already determined that the DCP might be more susceptible to ischemia than the SCP owing to anatomical differences [21]. The DCP leads into the superficial venules through vertical anastomoses, whereas the SCP directly connects to the retinal arterioles through transverse capillaries with higher perfusion pressures [22–24]. It might explain our results.

As we determined the parafoveal VD in four different areas (superior, temporal, inferior, and nasal), the changes in each area can be divided and compared. We proved that significant nasal parafoveal changes occur in the SCP and the DCP in SO tamponade eyes when compared with those in fellow control eyes. The average VD in the DCP was also significantly different between the groups. Several hypotheses could explain these changes. First, the change in OCTA parameters may reflect the microvascular changes in the papillomacular bundle (PMB), which is particularly more vulnerable to ischemic changes compared to other areas. Pellegrin et al. [25] reported a case of metabolic optic neuropathy where the decreased macular VDs corresponded to areas of the PMB. They demonstrated a reduction of VD not only at the peripapillary area but also at the macular area itself. Second, because the VD of the nasal parafoveal area in both the SCP and DCP was smaller than that of other areas, this area might be more sensitive to changes. However, more research is necessary to prove this hypothesis.

An enlarged FAZ area can represent microvascular alterations in the fovea. This has been previously reported in various ischemic retinal diseases, and it is related to poor visual outcomes [12, 26]. McKay et al. reported that significant microvascular changes were detected in the DCP after successful macula-off RD repair with SO or gas tamponade [13]. We also found enlarged FAZ in the eyes with SO tamponade when compared with that in controls; this was markedly observed in both the SCP and DCP (p = 0.043, p = 0.002, respectively). Nam at el. [12] suggested that this ischemic change may be caused by fluid movements through the retinal tear, which may lead to a hypoxic retina with diffuse vascular occlusions. Inflammatory cytokines and prostaglandins were elevated in the subretinal fluid. These findings can provide an insight into the ischemic microvascular alterations that occur after SO tamponade.

The safety issues and pathogenesis related to SO tamponade are still not completely understood. Retinal toxicity may be caused by the mechanical pressure of the SO, which blocks the exchange of oxygen between the vitreous and the retina, resulting in metabolic derangement of the retina [27]. Macular microcirculation has been shown to decrease significantly 1 month after SO injection [19]. Long-term SO tamponade can result in retinal arterial and venous changes, including arterial narrowing, that could be caused by impaired penetration of oxygen into the vitreous [28]. Another possible cause is the exposure of the retina to light during surgery [29, 30]. Yamada et al. [30] demonstrated that SO‐filled eyes can be damaged during SO removal surgeries because of SO having a higher refractive index. Nevertheless, light-induced retinal damage varies depending on not only the volume of SO but also the axial length and intraocular lens power. Increased exposure of the fovea to light during SO removal can induce photoreceptor damage.

In general, OCTA is used to detect subtle vascular alterations in patients with RRD treated with SO tamponade. According to our results, distinct changes can be observed in the nasal parafoveal area in patients with RRD treated with SO tamponade.

There are several limitations to our study. First, this study was a retrospective observational study. A longitudinal study should be required to explain the microvascular changes after a successful surgery with SO tamponade. Second, the sample size was relatively small to draw clear conclusions. Third, we acquired the main outcome measures at least 3 months after primary vitrectomy and 3 months after SO removal. This is a sufficient period to detect the improvement of post-surgical edema and inflammation. However, long-term follow-up studies after SO removal may reveal a recovery of vascular alterations. Finally, OCTA image artifacts can interfere with precise evaluation of retinal microvasculature because projection artifacts might disturb imagining of the deep layer. Further prospective studies would provide data to better understand changes in the vascular parameters and their relationship with functional and anatomical results.

## Conclusions

BCVA improved significantly following a successful retinal reattachment surgery. Baseline BCVA and the duration of SO tamponade were associated with postoperative BCVA. The average VD in the DCP and the VD of the nasal parafoveal area in both the DCP and SCP decreased relative to those in the control eye. The FAZ area in the DCP and SCP significantly increased when compared with that in the control eye. Our results suggest that SO tamponade could lead to negative microvascular changes, especially in the nasal parafoveal area of both the SCP and DCP. Moreover, the macular-off RRD group had a lower VD in the nasal parafoveal area of the DCP than the macular-on RRD group, potentially suggesting that macular involvement could influence the microvascular changes of the vessels. Further prospective studies would provide data to better understand these changes.

## Supporting information

S1 Fig (a, d) Fundus photography of before and after silicone oil removal. (b, c) Measurements of foveal avascular zone area and (e, f) results of vessel density calculations using optical coherence tomography angiography (superficial capillary plexus, deep capillary plexus).
(TIF)
