# Supplementary figures and images for: Microvascular changes on optical coherence tomography angiography after rhegmatogenous retinal detachment vitrectomy with silicone tamponade
Source: PLoS One. 2021 Mar 12;16(3):e0248433. doi: 10.1371/journal.pone.0248433 (PMC7954302; doi:10.1371/journal.pone.0248433)

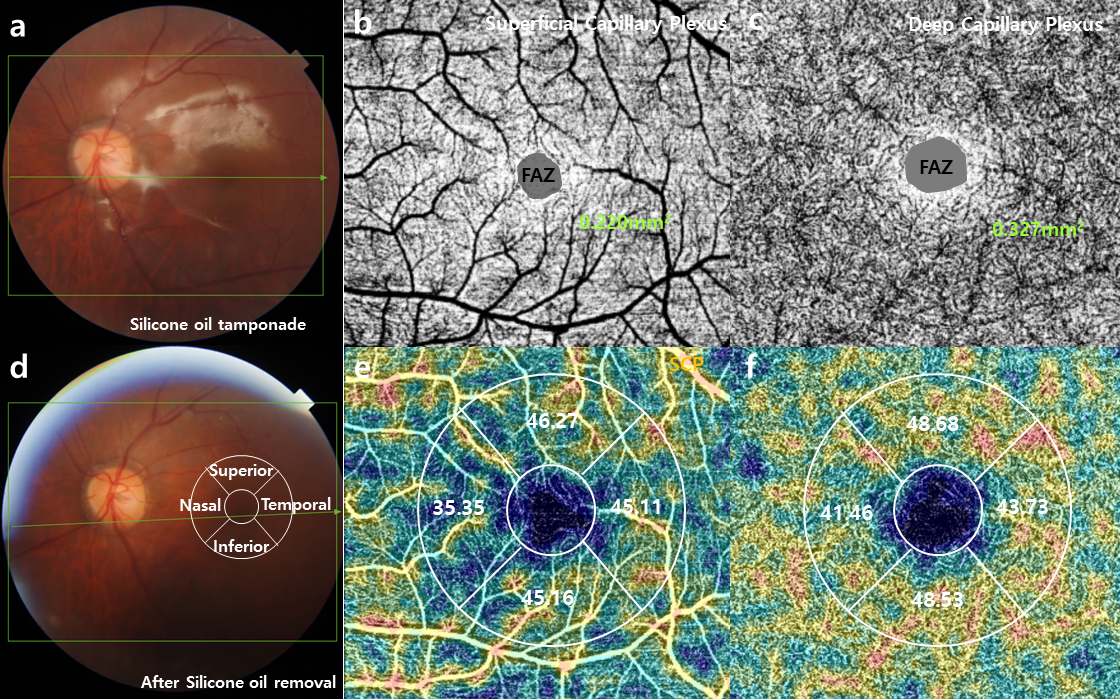

Supplement: S1 Fig — (a, d) Fundus photography of before and after silicone oil removal. (b, c) Measurements of foveal avascular zone area and (e, f) results of vessel density calculations using optical coherence tomography angiography (superficial capillary plexus, deep capillary plexus). (TIF) [file pone.0248433.s001.tif]
